# Significant dysregulation of lipid metabolism in patients with papillary thyroid carcinoma after thyroidectomy

**DOI:** 10.3389/fendo.2023.1223527

**Published:** 2023-10-12

**Authors:** Liang Zhou, Shuo Li, Yuqi Wu, Qianming Chen, Xiaotong Hu, Junchang Jiang, Yaoyao Shi, Dan Shen, Lei Xie

**Affiliations:** ^1^ Head and Neck Surgery, The affiliated Sir Run Run Shaw Hospital, Zhejiang University School of Medicine, Hangzhou, Zhejiang, China; ^2^ Key Laboratory of Digital Technology in Medical Diagnostics of Zhejiang Province, Dian Diagnostics Group Co., Ltd., Hangzhou, Zhejiang, China; ^3^ Department of Medical, Hangzhou Calibra Diagnostics Co., Ltd, Hangzhou, Zhejiang, China; ^4^ Stomatology Hospital, School of Stomatology, Zhejiang University School of Medicine, Zhejiang Provincial Clinical Research Center for Oral Diseases, Hangzhou, Zhejiang, China; ^5^ Pathology, The affiliated Sir Run Run Shaw Hospital, Zhejiang University School of Medicine, Hangzhou, Zhejiang, China

**Keywords:** papillary thyroid carcinoma, serum metabolomics, lipid metabolism, thyroidectomy, post-operative complications

## Abstract

**Introduction:**

Thyroidectomy and thyrotropin suppressive therapy is the widely used surgical treatment for papillary thyroid carcinoma (PTC) patients. However, systematic metabolic changes of post-operative PTC patients were rarely reported.

**Methods:**

Here, untargeted metabolomic detection of cohorts from PTC before (t0) and 1-month-after (t1) thyroidectomy, were performed to characterize circulating metabolic signatures after surgical treatment.

**Results:**

Our results showed PTC patients exhibited lower thyroid stimulating hormone degree, higher total thyroxine, and significant lipid-related metabolic alternations after thyroidectomy, which included 97 upregulations (including 93 lipids) and 5 downregulations (including 2 lipids and 3 nucleotides). Enrichment of metabolic pathways mainly included biosynthesis of fatty acids, purine metabolism, and linoleic acid metabolism. We also demonstrated that differential surgical approaches (hemi- and total thyroidectomy) and post-operative complication phenotypes (insomnia, fatigue), might lead to characteristic metabolic signatures.

**Discussion:**

This study revealed dynamic changes of metabolite characteristics of PTC patients after surgical treatment, which were associated with clinical thyroid function parameters, surgical approaches, and complication occurrence. It enlightened us to pay more attention on the post-operative metabolic dysregulation of PTC patients and their long-term qualities of life, so as to provide cautious clinical decisions on surgical choices, treatments, and follow-up details.

## 1 Introduction

Thyroid carcinoma is one of the most common endocrine malignancies, 85-90% of which were pathologically classified as papillary thyroid carcinoma (PTC) (1–3). The incidence of thyroid carcinoma has reached 9.61/10^5^ in 2015, and increased annually in China (4). Fortunately, for patients diagnosed with PTC, their 10-year overall survival (OS) rates were up to 98% in developed countries and above 90% in China (5–7), suggesting high efficiencies of PTC treatment. Due to the relatively longer OS and better prognosis of PTC, compared with other malignancies, it is necessary to take both treatment efficiency and long-term quality of life, into consideration, for clinicians.

Thyroidectomy is widely recommended for PTC treatment, in combined with levothyroxine suppressive therapy (or supplement) afterwards, to compromise the gland loss and to reduce the risk of recurrence (5, 8). In the previous perceptions, maintaining the normal levels of serum thyroid hormone (euthyroidism or subclinical hyperthyroidism) after surgery is considered enough to ensure normal physiological conditions and good long-term quality of life (5). However, many studies have found that the restoration of euthyroidism failed to ensure the restoration of quality of life (9–11). A study interviewed 146 thyroid cancer patients and have found that, about 50% of patients who received surgical operation, still reported chills and fatigues during thyroid hormone replacement treatment (12). Along with euthyroidism, subclinical hyperthyroidism is also associated with many adverse effects (13, 14), which might be associated with the endocrine and metabolic dysregulation after surgery (14–17). Therefore, a deep understanding towards systemic physiological changes after thyroidectomy would provide crucial information and contribute to improving patients’ quality of life.

Metabolomics comprised the entire set of small molecules generated by metabolic activity, carrying a great promise for understanding of biochemical pathway associated with disease development and prognosis (18–20). Erawijantari et al. (21) has revealed the gut metabolome changes after gastrectomy and possibly associated with post-operative comorbidities, which could benefit the patients in the future. Metabolomic studies focused on thyroid cancer have been conducted to classify pathological subtypes (22, 23), predict distant metastasis (24), and discover biomarkers for prognostic and diagnostic of thyroid carcinoma (19). For example, Gupta et al. (19) previously found that choline could be a potential biomarker for thyroid cancer diagnosis. Yao et al. (20) found significant differences in lipid metabolic pathways between benign thyroid nodules and malignant thyroid tumors. However, systematic metabolic pattern of post-operative PTC patients has not been reported yet.

In this study, we hypothesized that systematic metabolic profiles of PTC patients would be disrupted after thyroidectomy and thyrotropin suppressive therapy. Those molecular changes might be affected by the surgical approaches, and be associated with the occurrence of post-operative complications. To verify the hypothesis, paired serum samples were collected before and 1-month-after surgical treatment, for metabolomic detection and bioinformatic analysis, with the further aim of providing evidences for clinical decision making and quality of life maintenance.

## 2 Materials and methods

### 2.1 Patients and study design

Serum samples of the PTC patients (pathological confirmation) were paired-collected before (t0, n=50) and 1-month-after (t1, n=50) surgery. Patients were selected from the Department of Head and Neck Surgery in Sir Run Run Shaw Hospital, aged between 20 to 50 years old, both male and female.

The exclusion criterions were as follows: (1) patients with history of smoking or alcohol consumption during the enrolled period; (2) patients with abnormal liver or kidney function before surgery; (3) female patients who with or during menopause, or with pregnancy, during the enrolled period; (4) patients with sleep disorders or other definitive diagnostic psychologic disorders.

The surgical approach, thyroid function and other clinical parameters were recorded before surgery and during the follow-up period. Serum thyroid function levels of patients were detected with chemiluminescent immunoassay (CLIA) during clinical routines.

This study was approved by the Ethics Committee of Sir Run Run Shaw Hospital (No. KY202220811-73). Consent has been obtained from each patient after full explanation of the purpose and nature of all procedures used.

### 2.2 Serum untargeted metabolomic detection

Untargeted metabolomic detection was conducted by Calibra Lab at DIAN Diagnostics (Hangzhou, Zhejiang, China) on their CalOmics metabolomics platform. Sample processing was performed as previously described (22, 25). Briefly, the serum samples were mixed and extracted with methanol in a ratio of 1:4. After being shaken for 3 minutes, mixtures were centrifuged at 4000 × g for 10 minutes at room temperature. Supernatant were transferred to 4 sample plates, dried, re-dissolved, and finally injected into Ultra Performance Liquid Chromatography (UPLC) Tandem Mass Spectrometry (UPLC-MS/MS) systems, respectively. The instruments for the four UPLC-MS/MS methods were mentioned as previously described (22). Briefly, supernatant was divided into four fractions: two for analysis using two separate reverse-phase/ultra-performance liquid chromatography (RP/UPLC)-MS/MS methods with positive ion-mode electrospray ionization (ESI), one for analysis using RP/UPLC-MS/MS with negative-ion mode ESI, and one for analysis using hydrophilic interaction liquid chromatography (HILIC)/UPLC-MS/MS with negative-ion mode ESI.

### 2.3 Metabolomic data analysis

Raw data of UPLC-MS/MS were firstly processed with quality control. With the confirmation of quality control inspection, ion peaks were extracted with proprietary in-house hardware and software. Metabolites identification were performed by searching an in-house library as previously reported (22, 25). Identification of metabolites in samples requires strict matching of three criteria between experimental data and library entry: narrow window retention index (RI), accurate mass with variation less than 10 ppm and MS/MS spectra with high forward and reverse searching scores. Raw peak area of each metabolite was calculated with area-under-the-curve (AUC).

Raw peak area data were normalized before further statistical analysis. Briefly, raw peak areas were log-transformed (log2), normalized with median, and imputed missing values with minimal detection value of all samples, to finish the data normalization. Normalized data were further utilized for multivariate analysis to obtain differential metabolites among groups.

### 2.4 Statistics

All statistical analyses of metabolomic data were performed with R language (versions 4.1.0). Differential metabolites between t0 and t1 were found by parametric (Student’s t-test, ANOVA) or non-parametric (Kruskal-Wallis test) statistical methods, visualized with volcano plot. Adjusted-p < 0.05 was considered with statistically significance. Multivariate analysis approaches including principal component analysis (PCA) and orthogonal partial least square discriminant analysis (OPLS-DA), were also conducted. Variable importance in the projection (VIP) were generated in OPLS-DA. Correlation among clinical data and key metabolites were evaluated using Spearman’s correlation analysis. Enrichment analysis and pathway analysis were performed with MetaboAnalyst (https://www.metaboanalyst.ca/MetaboAnalyst/home.xhtml). Result visualization were provided with mixOmics and ggplot2 packages for scatter plot, pheatmap package for heatmaps.

Clinical data were analyzed with Chi-square test (qualitative variables, such as gender) or independent t-test for quantitative variables. Statistical analysis of clinical data was conducted with R, and p < 0.05 was considered statistically significant.

## 3 Results

### 3.1 Study design and enrolled patients’ characteristics

**Figure 1:**
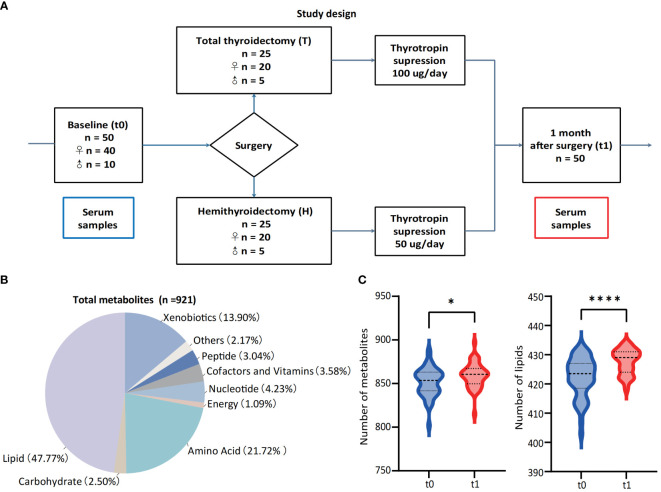
Study design and landscape of serum untargeted metabolomic detection. **(A)** Study design and paired collection of serum samples. **(B)** Classes of detected metabolic features from all 100 samples and the percentages of each class. **(C)** Numbers of metabolites and lipids before (t0) and 1-month-after (t1) surgery. *p < 0.05, **p < 0.01, ***p v 0.001, ****p < 0.0001.

This study was designed to provide insights into the metabolomic changes for PTC patients after thyroidectomy and 1-month thyrotropin suppression therapy. Thus, a total of 50 PTC patients (including 40 women and 10 men) were enrolled in this study, and serum samples were paired-collected before (t0) and 1-month-after (t1) surgery (**Figure 1A**). All the participants were pathologically confirmed as stage I of PTC and advised to undergo thyroidectomy according to the management guidline (5). The surgical approaches of patients were recorded as a basis for subgrouping (Hemithyroidectomy for H group, n = 25, and total thyroidectomy for T group, n = 25). The baseline clinical characteristics of participants were collected in **Table 1**, and their thyroid function indicators of t0 and t1 were shown in **Table 2**.

**Table 1:** Baseline characteristics of enrolled PTC participants.

	Overall (n = 50)
Sex	
F	40 (80.0%)
M	10 (20.0%)
**Age/year**	35.5 ± 6.21
**Body_Weight/kg**	59.9 ± 11.0
**BMI/kg × m^-2^**	22.3 ± 3.14
**Tumor_size/cm**	1.05 ± 0.730
Surgical_approach	
T	25 (50.0%)
H	25 (50.0%)
LN_dissection_central	
Y	49 (98.0%)
N	1 (2.0%)
LN_dissection_lateral	
Y	13 (26.0%)
N	37 (74.0%)
I131	
Y	10 (20.0%)
N	40 (80.0%)
Thyroiditis	
Y	19 (38.0%)
N	31 (62.0%)
Capsular_invation	
Y	19 (38.0%)
N	31 (62.0%)
Extrathyroidal_extension	
Y	11 (22.0%)
N	39 (78.0%)
Vascular_invation	
N	50 (100%)
Distant_metastases	
N	50 (100%)
T_stage	
T1a	24 (48.0%)
T1b	13 (26.0%)
T2	2 (4.0%)
T3b	11 (22.0%)
N_stage	
N0	15 (30.0%)
N1a	22 (44.0%)
N1b	13 (26.0%)
M_stage	
M0	50 (100%)
Stage_of_cancer	
I	50 (100%)

Values were shown as Mean ± S.D. or number (percentage).

F, female; M, male; T, total thyroidectomy; H, hemithyroidectomy; Y, yes; N, no.

**Table 2:** Changes in thyroid function levels of enrolled patients at baseline (t0) and after thyroidectomy plus one-month thyrotropin suppressive therapy (t1).

	t0 (n = 50)	t1 (n = 50)	P-value
TSH_degree mIU/L			
0-0.1	0 (0%)	10 (20.0%)	<0.001 ***
0.1-0.5	1 (2.0%)	12 (24.0%)	
0.5-2.0	37 (74.0%)	23 (46.0%)	
2.0-5.0	12 (24.0%)	1 (2.0%)	
5.0_above	0 (0%)	4 (8.0%)	
fT3 (1.71-3.71) pg/mL			
Normal	49 (98.0%)	47 (94.0%)	0.257
Above	0 (0%)	1 (2.0%)	
Below	0 (0%)	2 (4.0%)	
Missing	1 (2.0%)	0 (0%)	
tT3 (0.64-1.52) ng/mL			
Normal	50 (100%)	47 (94.0%)	0.241
Above	0 (0%)	0 (0%)	
Below	0 (0%)	3 (6.0%)	
fT4 (0.70-1.48) ng/dL			
Normal	48 (96.0%)	45 (90.0%)	0.251
Above	0 (0%)	2 (4.0%)	
Below	1 (2.0%)	3 (6.0%)	
Missing	1 (2.0%)	0 (0%)	
tT4 (4.87-11.72) μg/dL			
Normal	49 (98.0%)	41 (82.0%)	0.0234*
Above	0 (0%)	5 (10.0%)	
Below	1 (2.0%)	4 (8.0%)	
TgAb (0-4.11) IU/mL			
Normal	24 (48.0%)	21 (42.0%)	0.831
Above	25 (50.0%)	28 (56.0%)	
Missing	1 (2.0%)	1 (2.0%)	
TPOAb (0-5.61) IU/mL			
Normal	37 (74.0%)	37 (74.0%)	0.828
Above	12 (24.0%)	11 (22.0%)	
Missing	1 (2.0%)	2 (4.0%)	

Values were shown as Numbers (percentage).

TSH, thyrotropin; fT3, free triiodothyronine; tT3, total triiodothyronine; fT4, free thyroxine; tT4, total thyroxine; TgAb, anti-thyroglobulin; TPOAb, anti-thyroid peroxidase.

As **Table 1** showed, most of the included patients were female. Either the prophylactic or the therapy central neck dissections were performed in 49 of the 50 patients, and the therapy lateral neck dissections were admitted in 13 of 50 (26%) patients. Patients with extrathyroidal extension, vascular invasions, and multiple lymph node metastases would be advised to accept total thyroidectomy, and the AJCC TNM staging system was employed to evaluate the status of cancer (5). After thyroidectomy and thyrotropin suppression, participants showed lower thyroid stimulating hormone (TSH) degree and higher total thyroxine (tT4) level at t1.

### 3.2 Metabolomic changes in PTC patients after surgery

**Figure 2:**
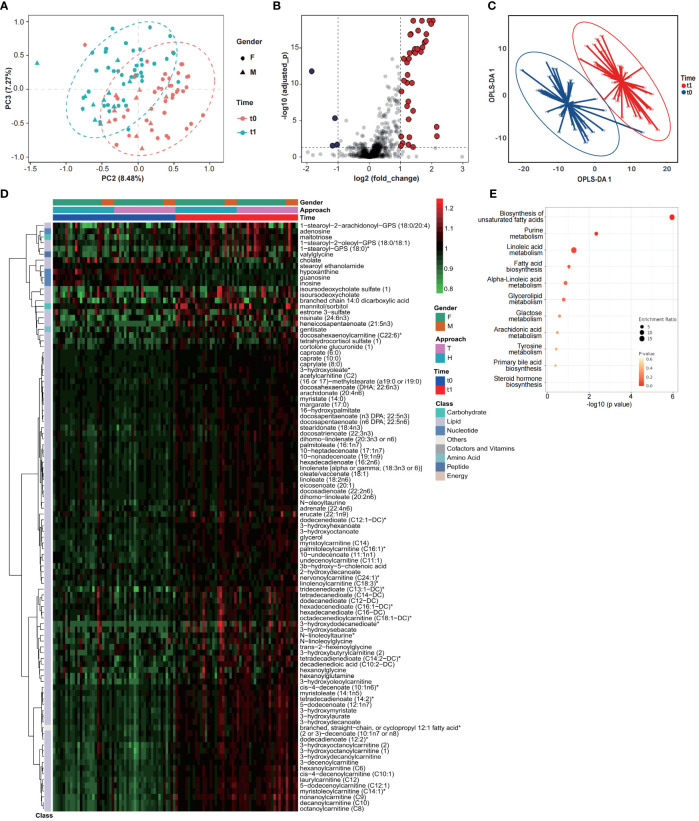
Serum metabolomic profiles of PTC patients significantly changed after thyroidectomy. Results of PCA **(A)**, volcano plot **(B)** and OPLS-DA **(C)** showed a significant structural change between t0 and t1. **(D)** Changing profiles were shown with 97 upregulated and 5 downregulated metabolites (OPLS-DA VIP>1.5, normalized data). **(E)** KEGG enrichment analysis of changing metabolites.

Considering of the metabolic regulation function of thyroid hormone, systematic metabolic dysregulation might exist after thyroidectomy. Paired-serum metabolome of 50 PTC patients at t0 and t1 were detected with UPLC-MS/MS, and a total of 921 metabolites were identified and quantified from 100 serum samples (**Figure 1B**). The number of serum metabolites detected in each sample, especially lipids, was significantly increased after thyroidectomy (**Figure 1C**). PCA and volcano plot both revealed the significant alternations of the serum metabolomic profile after thyroidectomy (**Figures 2A, B**).

To further identify the key metabolites, OPLS-DA was performed (**Figures 2C**; **S1**) and the metabolites contributed most to discriminate t0 and t1 samples (VIP > 1.5), considered as the key metabolomic changes after thyroidectomy, were summarized as a heatmap (**Figure 2D**; **Table S3**). These 102 key metabolites were classified into five main classes, as illustrated in **Figure 1D**, and 96 of them belongs to lipid, including varieties of fatty acids and fatty acid metabolism. 93 lipids were significantly increased when compared with t0, suggesting a dramatic dysregulation of lipid metabolism after thyroidectomy. Results of Kyoto Encyclopedia of Genes and Genomes (KEGG) enrichment analysis also exhibited significant enrichment of pathways related to fatty acid, including biosynthesis of unsaturated fatty acids, purine metabolism, linoleic acid metabolism and fatty acid biosynthesis (**Figure 2E**). Except for the lipid metabolism change, we also noticed that 3 nucleotide metabolites were significantly decreased at t1, including hypoxanthine, guanosine, and inosine, associated with purine metabolism (26).

### 3.3 Metabolites changing profiles of different surgical approaches

**Figure 3:**
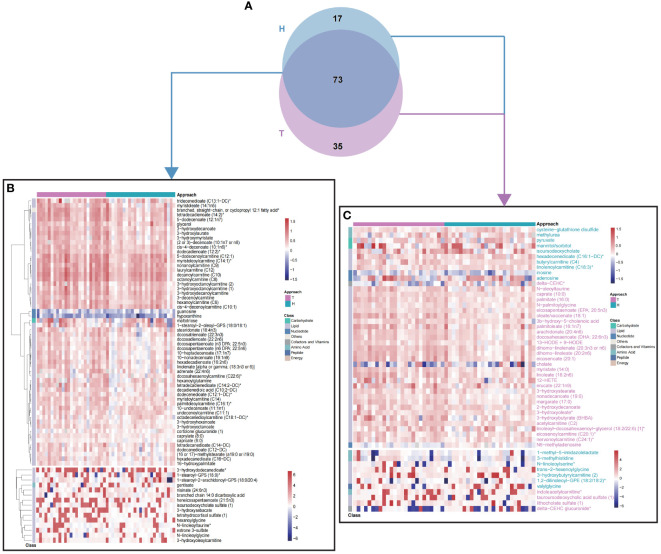
Differential fold-change profiles of T and H group between t0 and t1. **(A)** Venn plot of changing metabolites between t0 and t1, in T and H group, respectively. **(B)** 73 common metabolites contributed to discriminate t0 from t1, in different surgical approaches. **(C)** 52 differential metabolites contributed to discriminate t0 from t1, in different surgical approaches. Colors of metabolite names were consistent with colors of surgical approach. T, total thyroidectomy; H, hemithyroidectomy. Values of heatmap were shown with log2(fold change).

In order to test the hypothesis that different surgical approaches might cause different metabolomic impacts, patients were re-grouped to hemi- (H group) or total (T group) thyroidectomy subgroups. The thyroid function levels of H and T groups were listed in **Table S1**. And post-operative metabolomic changing profiles were analyzed with OPLS-DA as above (VIP >1.5). Briefly, 108 metabolites were different between t0 and t1 in T group (**Figure S2**, **Table S4**) and 90 metabolites significantly changed after hemithyroidectomy (**Figure S3**, **Table S5**). Majority of these metabolites were lipids and were consistent with findings in **Figure 2D**, which confirmed the dysregulation of lipid metabolism after surgery. Further comparison between these two sets of changed metabolites demonstrated that while a great portion of these metabolites were identical between T and H group (**Figure 3B**), there were still unique metabolomic changes as shown in Venn plot (**Figure 3A**) and heatmap (**Figure 3C**), with 17 metabolites in H group and 35 in T group, respectively. These results suggested that different surgical approaches might lead to different metabolism changes.

### 3.4 Lipid metabolites significantly associated with thyroid function

**Figure 4:**
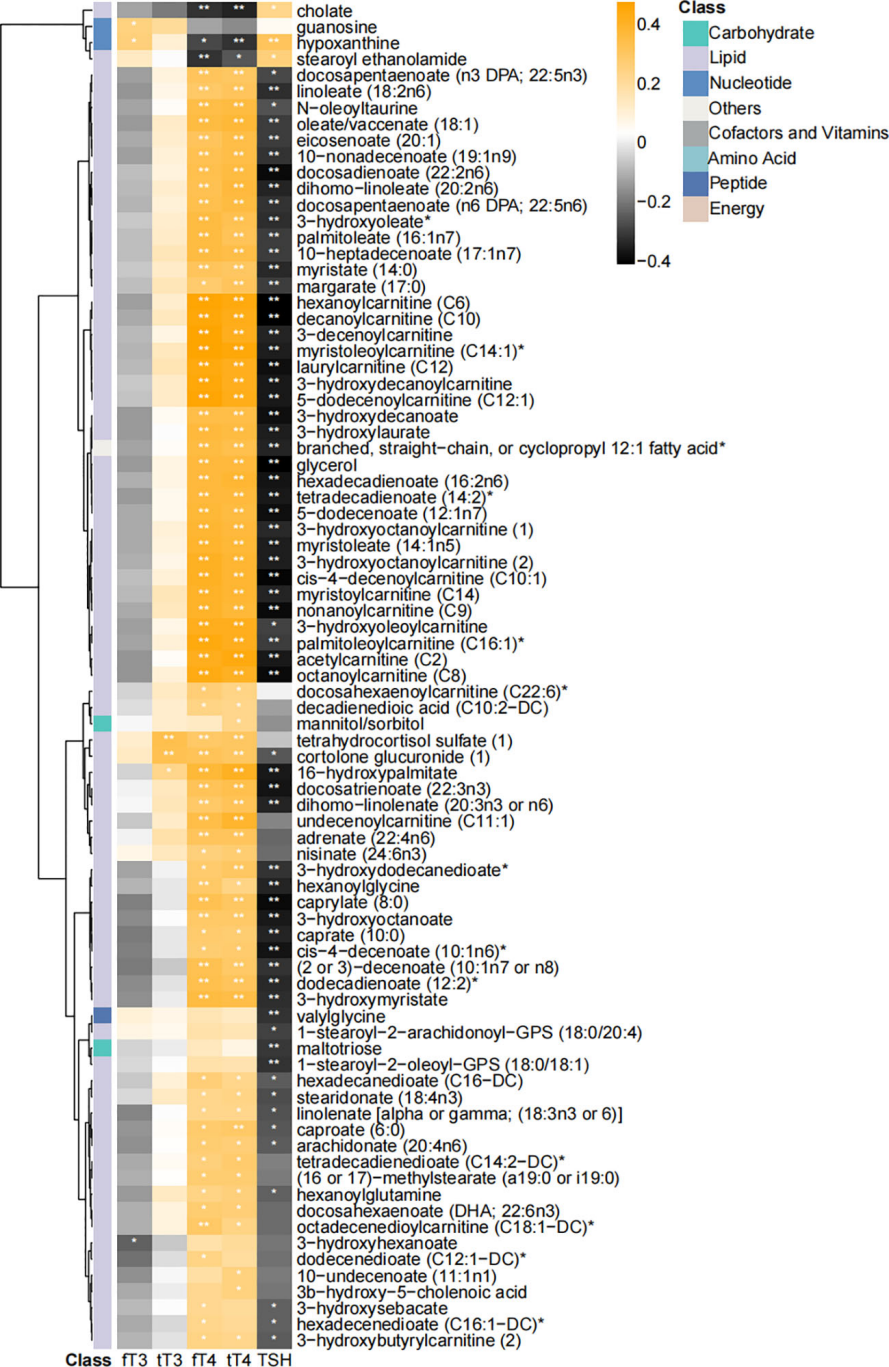
Heatmap of correlation coefficients among differential metabolites and thyroid function parameters. Correlation coefficients were calculated with Spearman’s correlation analysis. “*” and “**” represented *p* < 0.05 and *p* < 0.01, respectively. 83 of 102 were significantly correlated with thyroid function parameters.

Thyroid hormones (including triiodothyronine and thyroxine) are key regulators of lipid homeostasis. Thus, we further analyzed the correlation among thyroid function parameters and key differential metabolites by using Spearman’s correlation analysis (**Figure 4**). 83 of 102 metabolites, mainly lipids, showed significant correlation with thyroxine and thyroid-stimulating hormone (TSH) levels. The heatmap of the correlation coefficient showed that free thyroxine (fT4) and tT4 were positively correlated with most of the lipid-associated metabolites, and TSH level showed negative correlation with these metabolites (**Figure 4**).

Thus, to test the possibility that thyroid hormone dysregulation is responsible for the metabolism dysregulation after thyroidectomy, we compared the metabolomic profiles of patients with normal free triiodothyronine (fT3) and fT4 levels at t0 and t1 (8 patients were excluded because of the abnormal fT3 or fT4 levels, **Table S1**). Of 101 selected metabolites (OPLS-DA VIP >1.5, **Figure S4**), 94 were consistent with key metabolites in **Figure 2D**. Thus, thyroidectomy would remarkably affect the metabolic profile of PTC patients, even achieved normal thyroid function with medical supplement.

### 3.5 Metabolomic changing patterns between patients with and without post-operative complications

From our clinical observations and scientific publications, post-operative patients with routine thyrotropin suppression (5) might meet the TSH targets while still occurred complications associated with subclinical hyperthyroidism, such as physical tiredness and insomnia (23). In this study, according to chief complaints of patients, 12 of 50 enrolled patients occurred fatigue, and 9 of 50 suffered from insomnia at t1. Considering the abnormal thyroid function might contribute to the occurrence of post-operative complications (**Table S2**), only patients with normal thyroid function were included for complication-occurrence analysis (3 patients with abnormal fT3 or fT4 were excluded).

**Figure 5:**
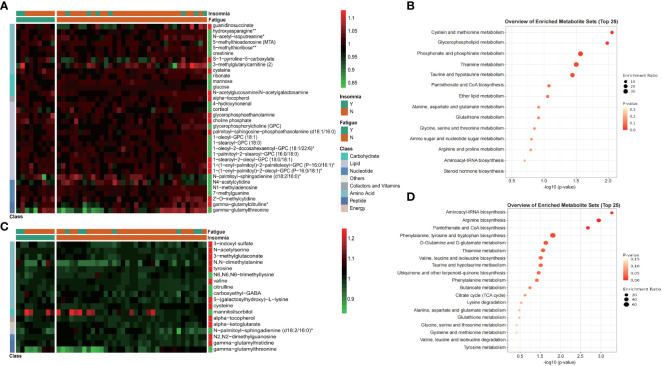
Metabolites exhibited differential distribution between those with and without postoperative complications, such as fatigue and insomnia. Heatmap and KEGG enrichment analysis of differential metabolites distribution (Wilcoxon test, *p*<0.01) between those with fatigue and non-fatigue **(A, B)**, or between those with insomnia and non-insomnia **(C, D)**. Values of heatmap were shown as normalized data. Y, yes; N, no.

Differential metabolites between those with (Y) and without (N) insomnia or fatigue were exhibited in **Figure 5** (Wilcoxon test, *p*<0.01). Totally, 34 metabolites were significantly different between patients suffered from fatigue or not (**Figures 5A, B**), especially amino acids associated with cysteine and methionine metabolism, such as 5-methylthioadenosine (MTA) and cysteine, and lipids associated with glycerophospholipid metabolism, phosphonate and phosphinate metabolism, such as glycerophosphorylcholine (GPC) and choline phosphate. As for occurrence of post-surgical insomnia, 18 significant changing metabolites were identified, including tyrosine, valine, and cysteine, associated with biosynthesis of aminoacyl-tRNA and varieties of amino acids (**Figures 5C, D**).

## 4 Discussion

Thyroidectomy is widely used and recommended for PTC treatment. With the purpose of reducing the risk of recurrence, thyrotropin suppression therapy was also recommended. However, post-operative complications, such as hormone disturbance and physical tiredness, are constantly reported, suggesting a metabolic disturbance after surgery. In this study, we provided the first description of the metabolic change after thyroidectomy, by comparing the serum metabolome between pre- and post-surgery samples. Results showed that over a hundred metabolites were dysregulated after surgery, demonstrated the occurrence of a significant metabolism dysregulation caused by thyroidectomy.

PCA and OPLS-DA models showed distinctively differential profiles of metabolites, between t0 and t1, demonstrating the strong influence of surgery, which could not be compensate by oral supplement of levothyroxine. The anesthesia and surgical wound would obviously affect the metabolism, immediately, after operation. When the time point came to 1-month-after surgery, the metabolic change should be mainly contributed to the gland loss and hormone changes rather than the immediate surgical effect. Our findings about increase of multiple lipid metabolites, involved in multiple dicarboxylate or acyl-glutamine fatty acid metabolism, glycogen metabolism and glycerolipid metabolism, revealed the increase of fatty acid beta- and omega-oxidation activity (24, 25, 27, 28). Our results showed similar perturbed pathway profiles compared with the former publication (29, 30). In addition, the increase of plasma free fatty acid level is not only a sign of energy metabolism reprograming, but also a modifiable risk factor for cardiovascular disease and considered an essential link in the onset of endothelial dysfunction in cardiovascular diseases (31, 32). Interestingly, there are reports demonstrating that thyroid cancer patients treated with thyroidectomy were at an increased risk of cardiovascular diseases (33, 34). Although the phenotype of cardiovascular disease was not been focused in this study, our findings might provide supporting evidence for cardiovascular risk caused by thyroidectomy, as the removal of thyroid gland led to increase fatty acid level, even with levothyroxine supplementation.

Thyroid hormones, including T3 and T4, are central modulating components of the hypothalamic-pituitary-thyroid axis, involved in a broad range of physiological pathways, from energy expenditure to lipid metabolism (30). Results of Spearman’s correlation analysis between serum thyroid function indicators and the differential metabolites (mainly lipid metabolites), showed the significant association between fT4, tT4 and lipid metabolites, especially with acyl-carnitines (**Figure 4**) in PTC patients after thyroidectomy. Those association also found in the serum euthyroid population (35), described as “higher serum fT4 levels are associated with increased serum acyl-carnitines concentrations”. Therefore, our findings further supported the positive correlation between T4 and serum acyl-carnitines, and raised the question that if these dysregulation of fatty acids after surgery is caused by thyroid hormone dysregulation. However, when making comparisons between t0 and t1 in normal thyroid function patients (**Figure S4**), majority of fatty acids were still found to be dysregulated, indicating that the metabolic changes were mainly caused by thyroidectomy, which reminded us that only focusing on hormone levels was not enough to ensure a healthy future for PTC patients received thyroidectomy, and a more systematic evaluating system should be developed in the future.

Our study also revealed that different surgical approaches, hemi- or total thyroidectomy, have largely similar but still distinct metabolomic influences (**Figure 3C**). Most metabolites showed consistent increasing or decreasing trend but inconsistent fold change, such as 3-hydroxybutyrate (BHBA), showed more increase in T group patients when compared with H group. As an ending product of ketogenesis and a marker of fatty acid beta oxidation (25, 36), the different changes of BHBA between T and H groups suggested that patients receiving total thyroidectomy might stimulate a stronger fatty acid metabolism comparing with those of hemithyroidectomy. Besides, changing characteristics of inosine and adenosine between T and H, contributed to differential purine metabolism (26), further indicating a dissimilar influence of surgical approach. In a word, our findings clearly revealed that hemi- and total thyroidectomy have different metabolomic impacts on PTC patient, which provided valuable information for future evaluation of different surgery approaches.

Fatigue and insomnia are two common long-term complications of thyroidectomy, and from our clinical routine of post-surgery attendant, some patients complained of post-operative fatigue and insomnia. Previous publications also reported kidney injury after thyroidectomy (37) and some metabolites were associated with cancer-related renal diseases (**Table S6**). Our findings showed that metabolites associated with fatigue (**Figure 5A**) were enriched into set of cysteine and methionine metabolism, with changes of 5-methylthioadenosine (MTA) and cysteine. Studies focused on fatigue have also reported similar findings in other disease like cancer (38) and COVID-19 (39). In addition, downregulation of glycerophospholipids like GPC, 1-oleoyl-GPC and 1-stearoyl-GPC, which are also associated with fatigue (40), were observed in fatigue patients. As for the insomnia, research about aberrant metabolism of insomniacs found numbers of metabolic pathway, including aminoacyl-tRNA biosynthesis, pantothenate and CoA biosynthesis, and phenylalanine, tyrosine and tryptophan biosynthesis, were significantly different from control group (41, 42). Those results were consistent with observations in our study (**Figure 5D**), with upregulations of amino acids, including tyrosine, valine, and cysteine, confirming the link between the occurrence of insomnia and these metabolic pathways. Taken together, our findings demonstrated the metabolic characteristic associated with post-operative complications, which could be used as biomarker for patient monitoring and as possible targets for intervention.

While we have provided the first systematic analysis of metabolomic changes caused by thyroidectomy, several limitations were still existed in our study. First, the patients included in the current study are mainly woman, and the biased gender ratio prevented us from investigating the possible difference between woman and man. Second, the follow-up was only done in one month, thus the changes in the long-term were not demonstrated by the current study. Third, the sample size of patient with post-operative complications were relatively small and some phenotypes of post-operative complications were not focused. We hope that this study could bring insights to researchers and clinicians about the long-term qualities of life for patients with good prognosis.

## 5 Conclusion

In conclusion, this study presents a systematic metabolomic investigation of PTC patient cohort after thyroidectomy, characterized with the significant upregulation of lipid metabolism, including enrichment pathways of biosynthesis of fatty acids, purine metabolism and linoleic acid metabolism. What is more, the molecular pathways including cysteine and methionine metabolism, aminoacyl-tRNA biosynthesis, are demonstrated association with occurrence of post-operative complications, such as fatigue and insomnia. Nevertheless, our results also exhibit differential metabolic influences of hemi- and total thyroidectomies. In total, these findings provide evidences of systematic distributions after surgical treatment, and potential molecular explanation of post-operative complications. Our study may shed light on potential mechanism of post-operative complication occurrence and provide evidences for clinical decision making to achieve improvement of post-operative quality of life.

## Data availability statement

The raw data supporting the conclusions of this article will be made available by the authors, without undue reservation.

## Ethics statement

The studies involving humans were approved by Ethics Committee of Sir Run Run Shaw Hospital. The studies were conducted in accordance with the local legislation and institutional requirements. The participants provided their written informed consent to participate in this study.

## Author contributions

Conceptualization: DS and LX; Data curation, software, formal analysis, and visualization: SL, YW and LX; Funding acquisition and resources: LZ; Investigation and methodology: SL, QC, and JJ; Project administration and supervision: LX, XH, and YS; Validation: LZ and DS; Writing – original draft: SL and LZ; Writing – review & editing: LX, DS and YW. All authors read and approved the final manuscript.
