# Supplementary material for: Significant dysregulation of lipid metabolism in patients with papillary thyroid carcinoma after thyroidectomy
Source: Front Endocrinol (Lausanne). 2023 Oct 12;14:1223527. doi: 10.3389/fendo.2023.1223527 (PMC10602777; doi:10.3389/fendo.2023.1223527)
Supplement: Supplementary file 1 [file DataSheet_1.docx]

Supplementary Material

Significant dysregulation of lipid metabolism in patients with papillary thyroid carcinoma after thyroidectomy

Liang Zhou^1†^, Shuo Li^2†^, Yuqi Wu^2,3^, Qianming Chen^4^, Xiaotong Hu^5^, Junchang Jiang^5^, Yaoyao Shi^5^, Dan Shen^2*^, Lei Xie^1*^

*** Co-correspondence: Lei Xie & Dan Shen
xielsrrsh@zju.edu.cn (L.X.); shendan@dazd.cn (D.S.)**

# Supplementary Figures and Tables

## Supplementary Figures


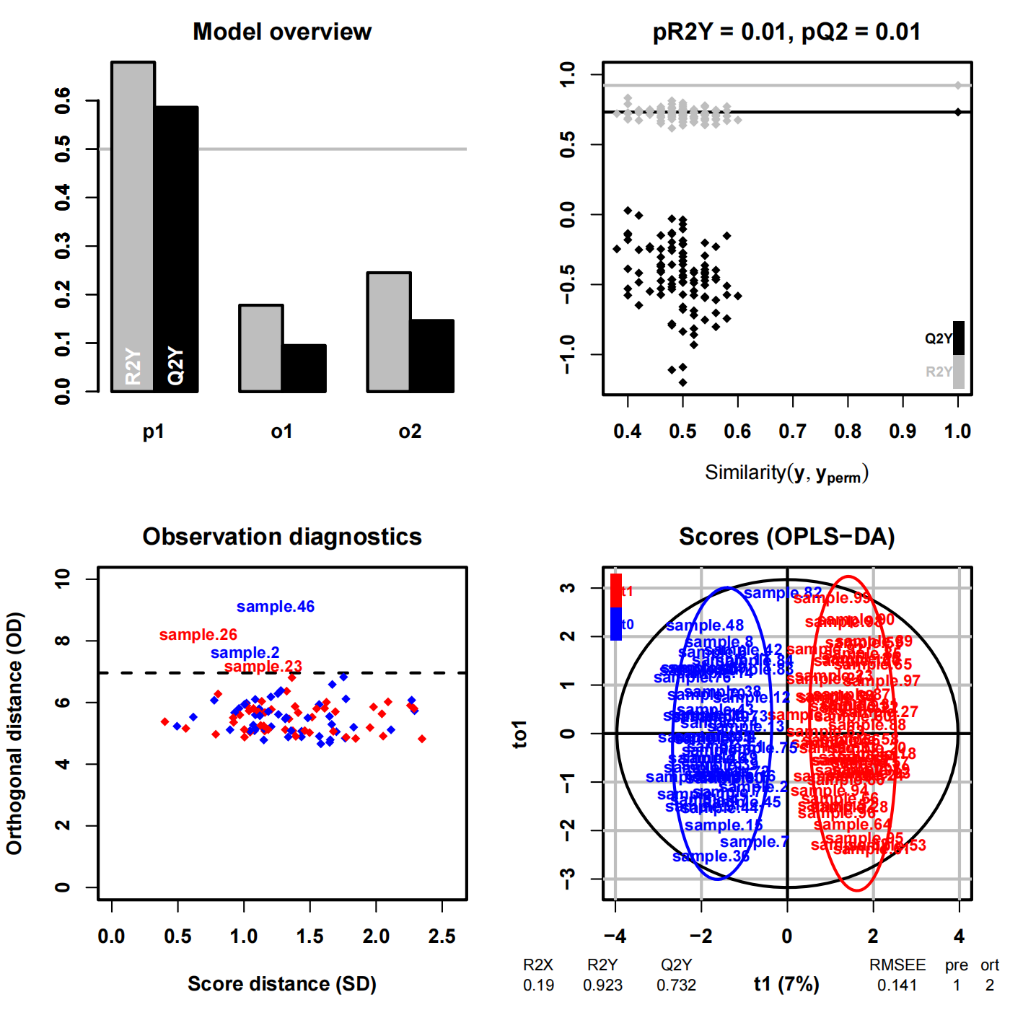


**Supplementary Figure 1.** **OPLS-DA model parameters to discriminate t0 and t1**.

**
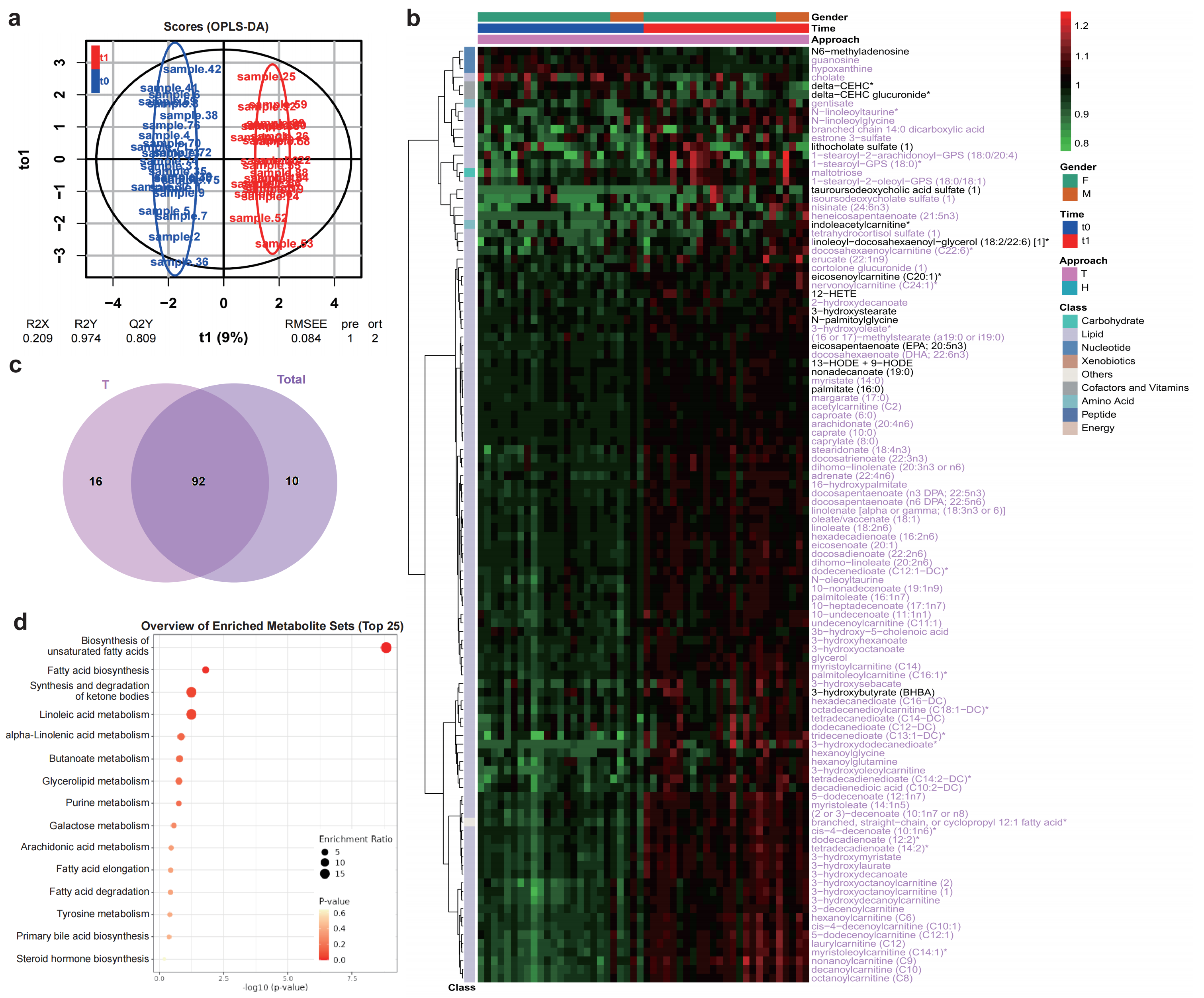
**

**Supplementary Figure 2.** **108 metabolites were significantly changed after total thyroidectomy (T group) at t1.** (a). OPLS-DA model parameters to discriminate t0 and t1 in T group (n = 25). (b). Heatmap of changing profiles in T group between t0 and t1. Metabolite names marked with purple were consistent with those of Figure 2d. (c). Venn plot of changing metabolites between T group (b) and total (Figure 2d). (d). Enrichment analysis of 108 metabolites.

**
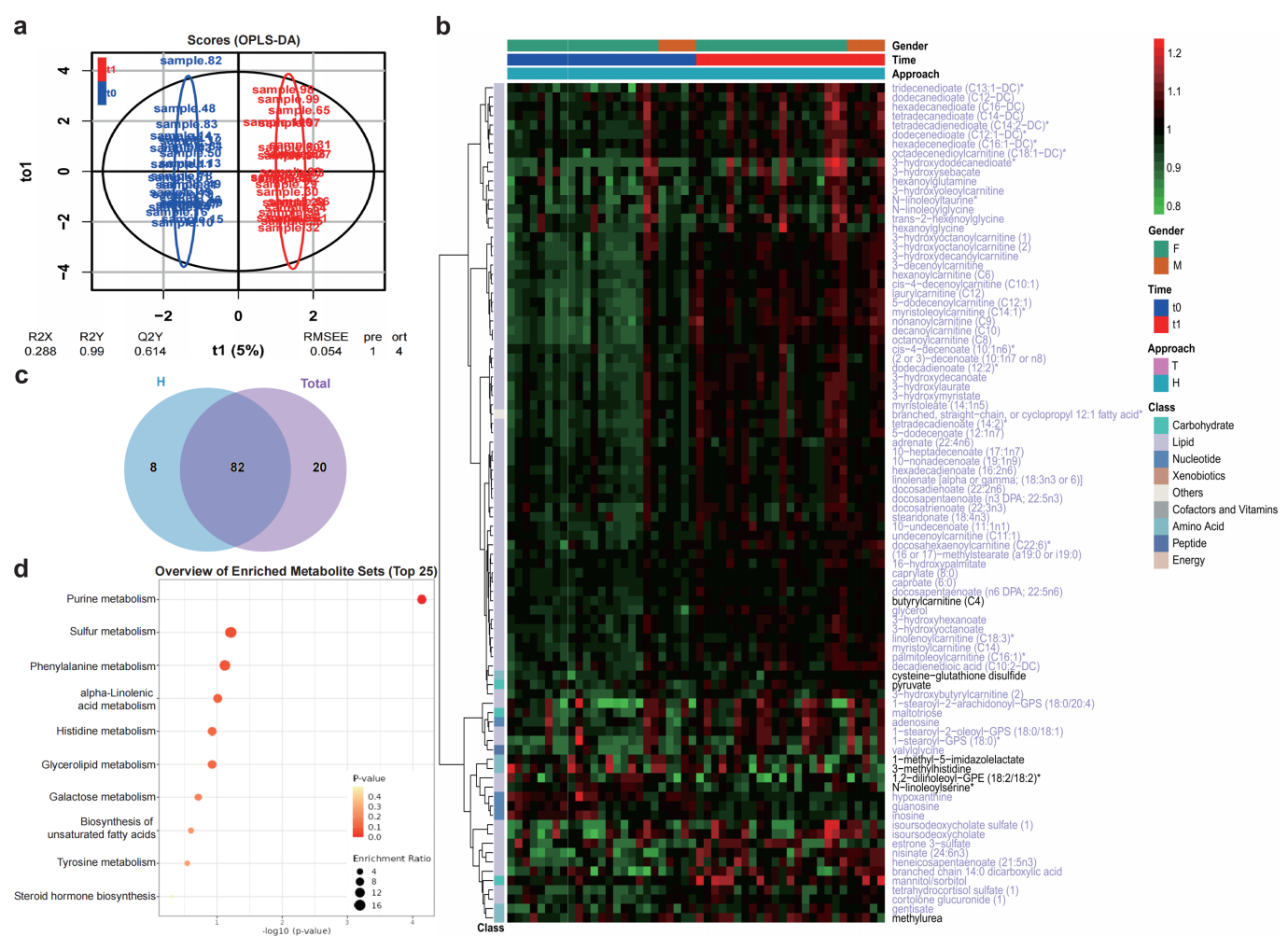
**

**Supplementary Figure 3.** **90 metabolites were significantly changed after hemithyroidectomy (H group) at t1.** (a). OPLS-DA model parameters to discriminate t0 and t1 in H group (n = 25). (b). Heatmap of changing profiles in H group between t0 and t1. Metabolite names marked with purple were consistent with those of Figure 2d. (c). Venn plot of changing metabolites between H group (b) and total (Figure 2d). (d). Enrichment analysis of 90 metabolites.

**Supplementary Figure 4.** **Metabolic changing profiles of patients with normal thyroid function parameters after thyroidectomy.**

## Supplementary Tables

**Supplementary Table 1.** **Differential distribution of thyroid function levels between patients with hemithyroidectomy (H group) and total thyroidectomy (T group), after surgery and one-month suppressive therapy (t1).**

|  | | **H group**  **(n = 25)** | | **T group**  **(n = 25)** | | **P-value** | |
| --- | --- | --- | --- | --- | --- | --- | --- |
| **TSH_degree mIU/L** | | |  | |  | |  |
|  | 0-0.1 | 0 (0%) | | 10 (40.0%) | | <0.001*** | |
|  | 0.1-0.5 | 4 (16.0%) | | 8 (32.0%) | |  |  |
|  | 0.5-2.0 | 21 (84.0%) | | 2 (8.0%) | |  |  |
|  | 2.0-5.0 | 0 (0%) | | 1 (4.0%) | |  |  |
|  | 5.0_above | 0 (0%) | | 4 (16.0%) | |  |  |
| **fT3 (1.71-3.71) pg/mL** | | | |  | |  | |
|  | Normal | 25 (100%) | | 22 (88.0%) | | 0.203 | |
|  | Above | 0 (0%) | | 1 (4.0%) | |  |  |
|  | Below | 0 (0%) | | 2 (8.0%) | |  |  |
| **tT3 (0.64-1.52) ng/mL** | | | |  | |  | |
|  | Normal | 25 (100%) | | 22 (88.0%) | | 0.234 | |
|  | Above | 0 (0%) | | 0 (0%) | |  |  |
|  | Below | 0 (0%) | | 3 (12.0%) | |  |  |
| **fT4 (0.70-1.48) ng/dL** | | | |  | |  | |
|  | Normal | 25 (100%) | | 20 (80.0%) | | 0.0622 | |
|  | Above | 0 (0%) | | 2 (8.0%) | |  |  |
|  | Below | 0 (0%) | | 3 (12.0%) | |  |  |
| **tT4 (4.87-11.72) μg/dL** | | | |  | |  | |
|  | Normal | 25 (100%) | | 16 (64.0%) | | 0.0041** | |
|  | Above | 0 (0%) | | 5 (20.0%) | |  |  |
|  | Below | 0 (0%) | | 4 (16.0%) | |  |  |
| **TgAb (0-4.11) IU/mL** | | | |  | |  | |
|  | Normal | 11 (44.0%) | | 10 (40.0%) | | 0.551 | |
|  | Above | 13 (52.0%) | | 15 (60.0%) | |  |  |
|  | Missing | 1 (4.0%) | | 0 (0%) | |  |  |
| **TPOAb (0-5.61) IU/mL** | | | |  | |  | |
|  | Normal | 19 (76.0%) | | 18 (72.0%) | | 0.241 | |
|  | Above | 4 (16.0%) | | 7 (28.0%) | |  |  |
|  | Missing | 2 (8.0%) | | 0 (0%) | |  |  |

**Supplementary Table 2. Distribution between post-operative complications and thyroid function parameters.**

|  | | **fT3** | |  | | **fT4** | | |  | |
| --- | --- | --- | --- | --- | --- | --- | --- | --- | --- | --- |
|  |  | **Normal**  **(n = 47)** | **Abnormal**  **(n = 3)** | | **P-value** | **Normal**  **(n = 45)** | **Abnormal (n = 5)** | **P-value** | |  |
| **Fatigue** | |  |  | |  |  |  |  | |  |
|  | Y | 11 (23.4%) | 1 (33.3%) | | 1.000 | 9 (20.0%) | 3 (60.0%) | 0.047* | |  |
|  | N | 36 (76.6%) | 2 (66.7%) | |  | 36 (80.0%) | 2 (40.0%) |  |  |  |
| **Insomnia** | |  |  | |  |  |  |  | |  |
|  | Y | 9 (19.1%) | 0 (0%) | | 0.696 | 9 (20.0%) | 0 (0%) | 0.269 | |  |
|  | N | 38 (80.9%) | 3 (100%) | |  | 36 (80.0%) | 5 (100%) |  |  |  |
| Abbreviation: Y, yes; N, no. | | | | | | | | | | |

**Supplementary Table 3.** **List of signature metabolites contributed to discriminating t0 with t1, for all enrolled patients (n = 50).**

| **Compound_name** | **HMDB_ID** | **SUPER_META_PATHWAY** | **OPLS-DA_VIP** |
| --- | --- | --- | --- |
| gentisate | HMDB0000152 | Amino Acid | 1.73 |
| maltotriose | HMDB0001262 | Carbohydrate | 2.17 |
| docosadienoate (22:2n6) | HMDB0061714 | Lipid | 2.14 |
| docosatrienoate (22:3n3) | HMDB0002823 | Lipid | 2.31 |
| N-linoleoyltaurine* |  | Lipid | 1.79 |
| N-oleoyltaurine |  | Lipid | 1.92 |
| estrone 3-sulfate | HMDB0001425 | Lipid | 1.81 |
| caprate (10:0) | HMDB0000511 | Lipid | 1.80 |
| 3-hydroxydodecanedioate* | HMDB0000413 | Lipid | 3.25 |
| 10-undecenoate (11:1n1) | HMDB0033724 | Lipid | 2.43 |
| tridecenedioate (C13:1-DC)* |  | Lipid | 2.96 |
| tetradecadienedioate (C14:2-DC)* |  | Lipid | 2.67 |
| glycerol | HMDB0000131 | Lipid | 2.83 |
| 1-stearoyl-GPS (18:0)* | HMDB0061698 | Lipid | 1.88 |
| tetrahydrocortisol sulfate (1) |  | Lipid | 1.63 |
| cis-4-decenoate (10:1n6)* | HMDB0004980 | Lipid | 2.97 |
| hexadecadienoate (16:2n6) | HMDB0000477 | Lipid | 2.43 |
| adrenate (22:4n6) | HMDB0002226 | Lipid | 2.08 |
| caprylate (8:0) | HMDB0000482 | Lipid | 2.16 |
| (2 or 3)-decenoate (10:1n7 or n8) |  | Lipid | 3.04 |
| docosapentaenoate (n3 DPA; 22:5n3) | HMDB0006528 | Lipid | 2.30 |
| oleate/vaccenate (18:1) | HMDB0000207 | Lipid | 1.76 |
| cortolone glucuronide (1) |  | Lipid | 1.53 |
| hexanoylglutamine |  | Lipid | 2.31 |
| caproate (6:0) | HMDB0000535 | Lipid | 1.76 |
| nisinate (24:6n3) | HMDB0002007 | Lipid | 2.62 |
| 3-hydroxysebacate | HMDB0000350 | Lipid | 2.50 |
| 3b-hydroxy-5-cholenoic acid | HMDB0000308 | Lipid | 1.58 |
| dodecadienoate (12:2)* |  | Lipid | 3.12 |
| 3-hydroxyhexanoate | HMDB0061652 | Lipid | 1.93 |
| trans-2-hexenoylglycine |  | Lipid | 1.52 |
| palmitoleate (16:1n7) | HMDB0003229 | Lipid | 1.78 |
| arachidonate (20:4n6) | HMDB0001043 | Lipid | 1.70 |
| docosahexaenoate (DHA; 22:6n3) | HMDB0002183 | Lipid | 1.65 |
| dihomo-linolenate (20:3n3 or n6) | HMDB0002925 | Lipid | 1.94 |
| heneicosapentaenoate (21:5n3) |  | Lipid | 2.40 |
| dihomo-linoleate (20:2n6) | HMDB0005060 | Lipid | 1.97 |
| branched chain 14:0 dicarboxylic acid |  | Lipid | 2.21 |
| 3-hydroxylaurate | HMDB0000387 | Lipid | 3.35 |
| eicosenoate (20:1) | HMDB0002231 | Lipid | 1.90 |
| 3-hydroxymyristate | HMDB0061656 | Lipid | 3.03 |
| decadienedioic acid (C10:2-DC) |  | Lipid | 1.77 |
| cholate | HMDB0000619 | Lipid | 1.82 |
| myristate (14:0) | HMDB0000806 | Lipid | 1.64 |
| hexadecanedioate (C16-DC) | HMDB0000672 | Lipid | 1.84 |
| stearidonate (18:4n3) | HMDB0006547 | Lipid | 2.16 |
| isoursodeoxycholate sulfate (1) |  | Lipid | 1.69 |
| linoleate (18:2n6) | HMDB0000673 | Lipid | 1.80 |
| isoursodeoxycholate | HMDB0000686 | Lipid | 1.67 |
| tetradecadienoate (14:2)* | HMDB0000560 | Lipid | 3.28 |
| 5-dodecenoate (12:1n7) | HMDB0000529 | Lipid | 3.28 |
| hexadecenedioate (C16:1-DC)* |  | Lipid | 1.61 |
| dodecenedioate (C12:1-DC)* | HMDB0000933 | Lipid | 2.33 |
| N-linoleoylglycine |  | Lipid | 1.70 |
| hexanoylglycine | HMDB0000701 | Lipid | 1.98 |
| erucate (22:1n9) | HMDB0002068 | Lipid | 1.76 |
| docosapentaenoate (n6 DPA; 22:5n6) | HMDB0001976 | Lipid | 2.16 |
| 3-hydroxydecanoate | HMDB0002203 | Lipid | 3.01 |
| tetradecanedioate (C14-DC) | HMDB0000872 | Lipid | 1.98 |
| 10-heptadecenoate (17:1n7) | HMDB0060038 | Lipid | 2.04 |
| 10-nonadecenoate (19:1n9) | HMDB0013622 | Lipid | 2.08 |
| myristoleate (14:1n5) | HMDB0002000 | Lipid | 2.70 |
| linolenate [alpha or gamma; (18:3n3 or 6)] | HMDB0003073 | Lipid | 1.95 |
| 3-hydroxyoctanoate | HMDB0001954 | Lipid | 2.44 |
| dodecanedioate (C12-DC) | HMDB0000623 | Lipid | 1.96 |
| margarate (17:0) | HMDB0002259 | Lipid | 1.61 |
| 2-hydroxydecanoate | HMDB0094656 | Lipid | 1.59 |
| (16 or 17)-methylstearate (a19:0 or i19:0) | HMDB0037397 | Lipid | 1.69 |
| 16-hydroxypalmitate | HMDB0006294 | Lipid | 1.97 |
| guanosine | HMDB0000133 | Nucleotide | 1.80 |
| hypoxanthine | HMDB0000157 | Nucleotide | 3.07 |
| inosine | HMDB0000195 | Nucleotide | 1.95 |
| branched-chain, straight-chain, or cyclopropyl 12:1 fatty acid* |  | Others | 3.37 |
| valylglycine | HMDB0029127 | Peptide | 1.52 |
| 3-hydroxyoleate* |  | Lipid | 1.71 |
| mannitol/sorbitol | HMDB0000765 | Carbohydrate | 1.64 |
| 3-hydroxyoctanoylcarnitine (1) | HMDB0061634 | Lipid | 3.01 |
| 3-hydroxyoctanoylcarnitine (2) |  | Lipid | 3.23 |
| 3-hydroxybutyrylcarnitine (2) | HMDB0013127 | Lipid | 1.81 |
| hexanoylcarnitine (C6) | HMDB0000756 | Lipid | 3.30 |
| acetylcarnitine (C2) | HMDB0000201 | Lipid | 1.68 |
| adenosine | HMDB0000050 | Nucleotide | 1.51 |
| stearoyl ethanolamide | HMDB0013078 | Lipid | 1.51 |
| octadecenedioylcarnitine (C18:1-DC)* |  | Lipid | 1.99 |
| 3-hydroxydecanoylcarnitine | HMDB0061636 | Lipid | 3.36 |
| 3-hydroxyoleoylcarnitine |  | Lipid | 3.01 |
| myristoylcarnitine (C14) | HMDB0254979 | Lipid | 2.73 |
| decanoylcarnitine (C10) | HMDB0000651 | Lipid | 3.79 |
| laurylcarnitine (C12) | HMDB0002250 | Lipid | 3.79 |
| octanoylcarnitine (C8) | HMDB0000791 | Lipid | 3.69 |
| nonanoylcarnitine (C9) | HMDB0013288 | Lipid | 3.88 |
| nervonoylcarnitine (C24:1)* | HMDB0006509 | Lipid | 1.55 |
| cis-4-decenoylcarnitine (C10:1) | HMDB0240585 | Lipid | 3.30 |
| undecenoylcarnitine (C11:1) |  | Lipid | 2.49 |
| 3-decenoylcarnitine |  | Lipid | 3.12 |
| palmitoleoylcarnitine (C16:1)* | HMDB0013207 | Lipid | 2.35 |
| 5-dodecenoylcarnitine (C12:1) | HMDB0013326 | Lipid | 3.63 |
| myristoleoylcarnitine (C14:1)* | HMDB0240588 | Lipid | 3.60 |
| linolenoylcarnitine (C18:3)* |  | Lipid | 1.55 |
| docosahexaenoylcarnitine (C22:6)* |  | Lipid | 1.81 |
| 1-stearoyl-2-arachidonoyl-GPS (18:0/20:4) | HMDB0012383 | Lipid | 2.08 |
| 1-stearoyl-2-oleoyl-GPS (18:0/18:1) | HMDB0010163 | Lipid | 1.92 |

**Supplementary Table 4.** **List of key metabolites contributed to discriminating t0 with t1, for patients with total thyroidectomy (T group, n = 25).**

| **Compound_name** | **HMDB_ID** | **SUPER_META_**  **PATHWAY** | **OPLS-DA_VIP** |
| --- | --- | --- | --- |
| gentisate | HMDB0000152 | Amino Acid | 1.67 |
| maltotriose | HMDB0001262 | Carbohydrate | 2.27 |
| delta-CEHC* |  | Cofactors and Vitamins | 1.66 |
| delta-CEHC glucuronide* |  | Cofactors and Vitamins | 1.86 |
| docosadienoate (22:2n6) | HMDB0061714 | Lipid | 2.27 |
| docosatrienoate (22:3n3) | HMDB0002823 | Lipid | 2.61 |
| N-linoleoyltaurine* |  | Lipid | 1.63 |
| N-oleoyltaurine |  | Lipid | 2.11 |
| estrone 3-sulfate | HMDB0001425 | Lipid | 1.82 |
| caprate (10:0) | HMDB0000511 | Lipid | 1.83 |
| 3-hydroxydodecanedioate* | HMDB0000413 | Lipid | 3.29 |
| 10-undecenoate (11:1n1) | HMDB0033724 | Lipid | 2.18 |
| tridecenedioate (C13:1-DC)* |  | Lipid | 2.94 |
| palmitate (16:0) | HMDB0000220 | Lipid | 1.65 |
| tetradecadienedioate (C14:2-DC)* |  | Lipid | 2.91 |
| glycerol | HMDB0000131 | Lipid | 2.63 |
| 1-stearoyl-GPS (18:0)* | HMDB0061698 | Lipid | 2.20 |
| N-palmitoylglycine | HMDB0013034 | Lipid | 1.60 |
| tetrahydrocortisol sulfate (1) |  | Lipid | 1.65 |
| cis-4-decenoate (10:1n6)* | HMDB0004980 | Lipid | 2.80 |
| hexadecadienoate (16:2n6) | HMDB0000477 | Lipid | 2.54 |
| eicosapentaenoate (EPA; 20:5n3) | HMDB0001999 | Lipid | 1.60 |
| adrenate (22:4n6) | HMDB0002226 | Lipid | 2.28 |
| caprylate (8:0) | HMDB0000482 | Lipid | 2.06 |
| (2 or 3)-decenoate (10:1n7 or n8) |  | Lipid | 2.94 |
| docosapentaenoate (n3 DPA; 22:5n3) | HMDB0006528 | Lipid | 2.52 |
| oleate/vaccenate (18:1) | HMDB0000207 | Lipid | 1.88 |
| cortolone glucuronide (1) |  | Lipid | 1.51 |
| hexanoylglutamine |  | Lipid | 2.42 |
| caproate (6:0) | HMDB0000535 | Lipid | 1.68 |
| nisinate (24:6n3) | HMDB0002007 | Lipid | 2.43 |
| 3-hydroxysebacate | HMDB0000350 | Lipid | 2.63 |
| 3b-hydroxy-5-cholenoic acid | HMDB0000308 | Lipid | 1.58 |
| dodecadienoate (12:2)* |  | Lipid | 2.89 |
| tauroursodeoxycholic acid sulfate (1) |  | Lipid | 1.65 |
| 3-hydroxyhexanoate | HMDB0061652 | Lipid | 1.82 |
| palmitoleate (16:1n7) | HMDB0003229 | Lipid | 1.80 |
| arachidonate (20:4n6) | HMDB0001043 | Lipid | 1.84 |
| docosahexaenoate (DHA; 22:6n3) | HMDB0002183 | Lipid | 1.82 |
| 13-HODE + 9-HODE | HMDB0004667 | Lipid | 1.58 |
| dihomo-linolenate (20:3n3 or n6) | HMDB0002925 | Lipid | 2.14 |
| heneicosapentaenoate (21:5n3) |  | Lipid | 1.95 |
| dihomo-linoleate (20:2n6) | HMDB0005060 | Lipid | 2.13 |
| branched chain 14:0 dicarboxylic acid |  | Lipid | 1.79 |
| 3-hydroxylaurate | HMDB0000387 | Lipid | 3.30 |
| eicosenoate (20:1) | HMDB0002231 | Lipid | 2.05 |
| 3-hydroxymyristate | HMDB0061656 | Lipid | 3.10 |
| decadienedioic acid (C10:2-DC) |  | Lipid | 1.98 |
| cholate | HMDB0000619 | Lipid | 1.71 |
| myristate (14:0) | HMDB0000806 | Lipid | 1.69 |
| hexadecanedioate (C16-DC) | HMDB0000672 | Lipid | 1.94 |
| stearidonate (18:4n3) | HMDB0006547 | Lipid | 2.23 |
| isoursodeoxycholate sulfate (1) |  | Lipid | 1.72 |
| linoleate (18:2n6) | HMDB0000673 | Lipid | 1.89 |
| lithocholate sulfate (1) |  | Lipid | 1.53 |
| tetradecadienoate (14:2)* | HMDB0000560 | Lipid | 3.23 |
| 5-dodecenoate (12:1n7) | HMDB0000529 | Lipid | 3.18 |
| dodecenedioate (C12:1-DC)* | HMDB0000933 | Lipid | 2.23 |
| N-linoleoylglycine |  | Lipid | 1.53 |
| 12-HETE | HMDB0006111 | Lipid | 1.81 |
| hexanoylglycine | HMDB0000701 | Lipid | 2.05 |
| erucate (22:1n9) | HMDB0002068 | Lipid | 1.81 |
| docosapentaenoate (n6 DPA; 22:5n6) | HMDB0001976 | Lipid | 2.25 |
| 3-hydroxydecanoate | HMDB0002203 | Lipid | 3.05 |
| tetradecanedioate (C14-DC) | HMDB0000872 | Lipid | 2.08 |
| 3-hydroxystearate | HMDB0010737 | Lipid | 1.69 |
| 10-heptadecenoate (17:1n7) | HMDB0060038 | Lipid | 2.14 |
| 10-nonadecenoate (19:1n9) | HMDB0013622 | Lipid | 2.18 |
| myristoleate (14:1n5) | HMDB0002000 | Lipid | 2.67 |
| linolenate [alpha or gamma; (18:3n3 or 6)] | HMDB0003073 | Lipid | 1.98 |
| 3-hydroxyoctanoate | HMDB0001954 | Lipid | 2.41 |
| nonadecanoate (19:0) | HMDB0000772 | Lipid | 1.52 |
| dodecanedioate (C12-DC) | HMDB0000623 | Lipid | 1.99 |
| margarate (17:0) | HMDB0002259 | Lipid | 1.83 |
| 2-hydroxydecanoate | HMDB0094656 | Lipid | 1.79 |
| (16 or 17)-methylstearate (a19:0 or i19:0) | HMDB0037397 | Lipid | 1.74 |
| 16-hydroxypalmitate | HMDB0006294 | Lipid | 2.13 |
| guanosine | HMDB0000133 | Nucleotide | 1.61 |
| hypoxanthine | HMDB0000157 | Nucleotide | 2.43 |
| branched-chain, straight-chain, or cyclopropyl 12:1 fatty acid* |  | Others | 3.32 |
| 3-hydroxyoleate* |  | Lipid | 1.82 |
| 3-hydroxybutyrate (BHBA) | HMDB0000011 | Lipid | 1.63 |
| 3-hydroxyoctanoylcarnitine (1) | HMDB0061634 | Lipid | 2.88 |
| 3-hydroxyoctanoylcarnitine (2) |  | Lipid | 3.13 |
| hexanoylcarnitine (C6) | HMDB0000756 | Lipid | 3.14 |
| acetylcarnitine (C2) | HMDB0000201 | Lipid | 1.76 |
| N6-methyladenosine | HMDB0004044 | Nucleotide | 1.53 |
| indoleacetylcarnitine* |  | Amino Acid | 1.51 |
| linoleoyl-docosahexaenoyl-glycerol (18:2/22:6) [1]* |  | Lipid | 1.53 |
| octadecenedioylcarnitine (C18:1-DC)* |  | Lipid | 1.85 |
| 3-hydroxydecanoylcarnitine | HMDB0061636 | Lipid | 3.12 |
| 3-hydroxyoleoylcarnitine |  | Lipid | 2.85 |
| myristoylcarnitine (C14) | HMDB0254979 | Lipid | 2.64 |
| decanoylcarnitine (C10) | HMDB0000651 | Lipid | 3.50 |
| laurylcarnitine (C12) | HMDB0002250 | Lipid | 3.47 |
| octanoylcarnitine (C8) | HMDB0000791 | Lipid | 3.37 |
| nonanoylcarnitine (C9) | HMDB0013288 | Lipid | 3.59 |
| eicosenoylcarnitine (C20:1)* |  | Lipid | 1.59 |
| nervonoylcarnitine (C24:1)* | HMDB0006509 | Lipid | 1.61 |
| cis-4-decenoylcarnitine (C10:1) | HMDB0240585 | Lipid | 2.95 |
| undecenoylcarnitine (C11:1) |  | Lipid | 2.18 |
| 3-decenoylcarnitine |  | Lipid | 2.73 |
| palmitoleoylcarnitine (C16:1)* | HMDB0013207 | Lipid | 2.23 |
| 5-dodecenoylcarnitine (C12:1) | HMDB0013326 | Lipid | 3.26 |
| myristoleoylcarnitine (C14:1)* | HMDB0240588 | Lipid | 3.36 |
| docosahexaenoylcarnitine (C22:6)* |  | Lipid | 1.81 |
| 1-stearoyl-2-arachidonoyl-GPS (18:0/20:4) | HMDB0012383 | Lipid | 1.88 |
| 1-stearoyl-2-oleoyl-GPS (18:0/18:1) | HMDB0010163 | Lipid | 1.96 |

**Supplementary Table 5.** **List of differential metabolites contributed to discriminating t0 with t1, for patients with hemithyroidectomy (H group, n = 25).**

| **Compound_name** | **HMDB_ID** | **SUPER_META_**  **PATHWAY** | **OPLS-DA_VIP** |
| --- | --- | --- | --- |
| 1-methyl-5-imidazolelactate |  | Amino Acid | 1.59 |
| gentisate | HMDB0000152 | Amino Acid | 1.87 |
| 3-methylhistidine | HMDB0000479 | Amino Acid | 1.81 |
| maltotriose | HMDB0001262 | Carbohydrate | 1.78 |
| docosadienoate (22:2n6) | HMDB0061714 | Lipid | 1.65 |
| docosatrienoate (22:3n3) | HMDB0002823 | Lipid | 1.68 |
| N-linoleoyltaurine* |  | Lipid | 1.91 |
| estrone 3-sulfate | HMDB0001425 | Lipid | 1.65 |
| 3-hydroxydodecanedioate* | HMDB0000413 | Lipid | 2.65 |
| 10-undecenoate (11:1n1) | HMDB0033724 | Lipid | 2.37 |
| tridecenedioate (C13:1-DC)* |  | Lipid | 2.66 |
| tetradecadienedioate (C14:2-DC)* |  | Lipid | 2.22 |
| glycerol | HMDB0000131 | Lipid | 2.98 |
| 1-stearoyl-GPS (18:0)* | HMDB0061698 | Lipid | 1.62 |
| N-linoleoylserine* |  | Lipid | 1.55 |
| tetrahydrocortisol sulfate (1) |  | Lipid | 1.83 |
| cis-4-decenoate (10:1n6)* | HMDB0004980 | Lipid | 2.80 |
| hexadecadienoate (16:2n6) | HMDB0000477 | Lipid | 2.06 |
| adrenate (22:4n6) | HMDB0002226 | Lipid | 1.66 |
| caprylate (8:0) | HMDB0000482 | Lipid | 1.96 |
| (2 or 3)-decenoate (10:1n7 or n8) |  | Lipid | 2.71 |
| docosapentaenoate (n3 DPA; 22:5n3) | HMDB0006528 | Lipid | 1.73 |
| cortolone glucuronide (1) |  | Lipid | 1.50 |
| hexanoylglutamine |  | Lipid | 1.81 |
| caproate (6:0) | HMDB0000535 | Lipid | 1.69 |
| nisinate (24:6n3) | HMDB0002007 | Lipid | 3.29 |
| 3-hydroxysebacate | HMDB0000350 | Lipid | 2.04 |
| dodecadienoate (12:2)* |  | Lipid | 3.07 |
| 3-hydroxyhexanoate | HMDB0061652 | Lipid | 1.91 |
| trans-2-hexenoylglycine |  | Lipid | 1.67 |
| heneicosapentaenoate (21:5n3) |  | Lipid | 2.88 |
| branched chain 14:0 dicarboxylic acid |  | Lipid | 2.67 |
| 3-hydroxylaurate | HMDB0000387 | Lipid | 2.89 |
| 3-hydroxymyristate | HMDB0061656 | Lipid | 2.44 |
| decadienedioic acid (C10:2-DC) |  | Lipid | 1.57 |
| hexadecanedioate (C16-DC) | HMDB0000672 | Lipid | 1.58 |
| stearidonate (18:4n3) | HMDB0006547 | Lipid | 1.90 |
| isoursodeoxycholate sulfate (1) |  | Lipid | 1.57 |
| isoursodeoxycholate | HMDB0000686 | Lipid | 1.72 |
| tetradecadienoate (14:2)* | HMDB0000560 | Lipid | 2.85 |
| 5-dodecenoate (12:1n7) | HMDB0000529 | Lipid | 2.89 |
| hexadecenedioate (C16:1-DC)* |  | Lipid | 1.72 |
| dodecenedioate (C12:1-DC)* | HMDB0000933 | Lipid | 2.10 |
| N-linoleoylglycine |  | Lipid | 1.86 |
| hexanoylglycine | HMDB0000701 | Lipid | 1.69 |
| docosapentaenoate (n6 DPA; 22:5n6) | HMDB0001976 | Lipid | 1.76 |
| 3-hydroxydecanoate | HMDB0002203 | Lipid | 2.48 |
| tetradecanedioate (C14-DC) | HMDB0000872 | Lipid | 1.52 |
| 10-heptadecenoate (17:1n7) | HMDB0060038 | Lipid | 1.64 |
| 10-nonadecenoate (19:1n9) | HMDB0013622 | Lipid | 1.61 |
| myristoleate (14:1n5) | HMDB0002000 | Lipid | 2.20 |
| linolenate [alpha or gamma; (18:3n3 or 6)] | HMDB0003073 | Lipid | 1.61 |
| 3-hydroxyoctanoate | HMDB0001954 | Lipid | 2.22 |
| dodecanedioate (C12-DC) | HMDB0000623 | Lipid | 1.59 |
| (16 or 17)-methylstearate (a19:0 or i19:0) | HMDB0037397 | Lipid | 1.55 |
| 16-hydroxypalmitate | HMDB0006294 | Lipid | 1.60 |
| guanosine | HMDB0000133 | Nucleotide | 2.07 |
| hypoxanthine | HMDB0000157 | Nucleotide | 3.29 |
| inosine | HMDB0000195 | Nucleotide | 2.62 |
| branched-chain, straight-chain, or cyclopropyl 12:1 fatty acid* |  | Others | 2.78 |
| valylglycine | HMDB0029127 | Peptide | 1.76 |
| pyruvate |  | Carbohydrate | 1.58 |
| mannitol/sorbitol | HMDB0000765 | Carbohydrate | 2.29 |
| 3-hydroxyoctanoylcarnitine (1) | HMDB0061634 | Lipid | 2.79 |
| 3-hydroxyoctanoylcarnitine (2) |  | Lipid | 3.00 |
| 3-hydroxybutyrylcarnitine (2) | HMDB0013127 | Lipid | 1.87 |
| hexanoylcarnitine (C6) | HMDB0000756 | Lipid | 3.23 |
| butyrylcarnitine (C4) | HMDB0002013 | Lipid | 1.65 |
| cysteine-glutathione disulfide | HMDB0000656 | Amino Acid | 1.61 |
| adenosine | HMDB0000050 | Nucleotide | 1.80 |
| methylurea |  | Amino Acid | 1.52 |
| octadecenedioylcarnitine (C18:1-DC)* |  | Lipid | 1.86 |
| 3-hydroxydecanoylcarnitine | HMDB0061636 | Lipid | 3.29 |
| 3-hydroxyoleoylcarnitine |  | Lipid | 2.92 |
| myristoylcarnitine (C14) | HMDB0254979 | Lipid | 2.54 |
| decanoylcarnitine (C10) | HMDB0000651 | Lipid | 3.84 |
| laurylcarnitine (C12) | HMDB0002250 | Lipid | 3.83 |
| octanoylcarnitine (C8) | HMDB0000791 | Lipid | 3.76 |
| nonanoylcarnitine (C9) | HMDB0013288 | Lipid | 3.89 |
| cis-4-decenoylcarnitine (C10:1) | HMDB0240585 | Lipid | 3.43 |
| undecenoylcarnitine (C11:1) |  | Lipid | 2.65 |
| 3-decenoylcarnitine |  | Lipid | 3.28 |
| palmitoleoylcarnitine (C16:1)* | HMDB0013207 | Lipid | 2.25 |
| 5-dodecenoylcarnitine (C12:1) | HMDB0013326 | Lipid | 3.71 |
| myristoleoylcarnitine (C14:1)* | HMDB0240588 | Lipid | 3.50 |
| linolenoylcarnitine (C18:3)* |  | Lipid | 1.68 |
| docosahexaenoylcarnitine (C22:6)* |  | Lipid | 1.80 |
| 1,2-dilinoleoyl-GPE (18:2/18:2)* | HMDB0009093 | Lipid | 1.66 |
| 1-stearoyl-2-arachidonoyl-GPS (18:0/20:4) | HMDB0012383 | Lipid | 2.35 |
| 1-stearoyl-2-oleoyl-GPS (18:0/18:1) | HMDB0010163 | Lipid | 1.81 |

**Supplementary Table 6. Summary of signature metabolites found in this study associated with thyroid carcinoma or cancer-related renal diseases, according to the literature research.**

| **Signature metabolites**  **in this study** | **Disease-outcome association on previous publications*** | |
| --- | --- | --- |
|  | **Thyroid carcinoma-associated** | **Cancer-related renal diseases-associated** |
| Metabolites associated with thyroidectomy  (t0 vs t1, n = 126) | Glycerol(5),  guanosine(1, 7),  hypoxanthine(1, 7, 8, 15),  inosine(7, 15, 16),  mannitol/sorbitol(13, 14), acetylcarnitine(11, 17),  adenosine(1),  decanolcarnitine(12),  pyruvate(1),  1-stearoyl-2-arachidonoyl-GPS (18:0/20:4)(9). | Adenosine(20),  octanoylcarnitine(18),  pyruvate(22). |
| Metabolites associated with the surgical approaches  (T vs H, n = 52) | Pyruvate(1),  mannitol/sorbitol(13, 14),  inosine(7, 15, 16),  acetylcarnitine(11, 17). | Adenosine(20). |
| Metabolites associated with  post-operative complications (including fatigue and insomnia,  n = 48) | Creatinine(5, 8),  cysteine(10),  mannose(13, 16),  glucose(3, 13, 16),  cortisol(1),  choline phosphate(7, 14, 15),  tyrosine(2, 6, 7),  valine(2, 4, 10),  citrulline(1, 11),  mannitol/sorbitol(13, 14),  alpha-ketoglutarate(10). | Creatinine(18, 20),  glucose(20),  cortisol(18),  3-indoxyl sulfate(18),  tyrosine(19, 21),  valine(22). |

* Referential publications were listed at the end of Supplementary materials.

**Supplementary references**

1. Cararo Lopes E, Sawant A, Moore D, Ke H, Shi F, Laddha S, et al. Integrated metabolic and genetic analysis reveals distinct features of human differentiated thyroid cancer. Clin Transl Med. 2023;13(6):e1298.

2. Metere A, Graves CE, Chirico M, Caramujo MJ, Pisanu ME, Iorio E. Metabolomic Reprogramming Detected by 1H-NMR Spectroscopy in Human Thyroid Cancer Tissues. Biology (Basel). 2020;9(6):112.

3. Zhou Q, Zhang LY, Xie C, Zhang ML, Wang YJ, Liu GH. Metabolomics as a potential method for predicting thyroid malignancy in children and adolescents. Pediatr Surg Int. 2020;36(2):145-153.

4. Shen CT, Zhang Y, Liu YM, Yin S, Zhang XY, Wei WJ, et al. A distinct serum metabolic signature of distant metastatic papillary thyroid carcinoma. Clin Endocrinol (Oxf). 2017;87(6):844-852.

5. Wojtowicz W, Zabek A, Deja S, Dawiskiba T, Pawelka D, Glod M, et al. Serum and urine 1H NMR-based metabolomics in the diagnosis of selected thyroid diseases. Sci Rep. 2017;7(1):9108.

6. Rezig L, Servadio A, Torregrossa L, Miccoli P, Basolo F, Shintu L, et al. Diagnosis of post-surgical fine-needle aspiration biopsies of thyroid lesions with indeterminate cytology using HRMAS NMR-based metabolomics. Metabolomics. 2018;14(10):141.

7. Seo JW, Han K, Lee J, Kim EK, Moon HJ, Yoon JH, et al. Application of metabolomics in prediction of lymph node metastasis in papillary thyroid carcinoma. PLoS One. 2018;13(3):e0193883.

8. Wu S, Tan G, Dong X, Zhu Z, Li W, Lou Z, et al. Metabolic profiling provides a system understanding of hypothyroidism in rats and its application. PLoS One. 2013;8(2):e55599.

9. Jiang N, Zhang Z, Chen X, Zhang G, Wang Y, Pan L, et al. Plasma Lipidomics Profiling Reveals Biomarkers for Papillary Thyroid Cancer Diagnosis. Front Cell Dev Biol. 2021;9:682269.

10. Abooshahab R, Hooshmand K, Razavi SA, Gholami M, Sanoie M, Hedayati M. Plasma Metabolic Profiling of Human Thyroid Nodules by Gas Chromatography-Mass Spectrometry (GC-MS)-Based Untargeted Metabolomics. Front Cell Dev Biol. 2020;8:385.

11. Farrokhi Yekta R, Rezaei Tavirani M, Arefi Oskouie A, Mohajeri-Tehrani MR, Soroush AR, Akbarzadeh Baghban A. Serum-based metabolic alterations in patients with papillary thyroid carcinoma unveiled by non-targeted 1H-NMR metabolomics approach. Iran J Basic Med Sci. 2018;21(11):1140-1147.

12. Huang FQ, Li J, Jiang L, Wang FX, Alolga RN, Wang MJ, et al. Serum-plasma matched metabolomics for comprehensive characterization of benign thyroid nodule and papillary thyroid carcinoma. Int J Cancer. 2019;144(4):868-876.

13. Shang X, Zhong X, Tian X. Metabolomics of papillary thyroid carcinoma tissues: potential biomarkers for diagnosis and promising targets for therapy. Tumour Biol. 2016;37(8):11163-75.

14. Farrokhi Yekta R, Rezaie Tavirani M, Arefi Oskouie A, Mohajeri-Tehrani MR, Soroush AR. The metabolomics and lipidomics window into thyroid cancer research. Biomarkers. 2017;22(7):595-603.

15. Tian Y, Nie X, Xu S, Li Y, Huang T, Tang H, et al. Integrative metabonomics as potential method for diagnosis of thyroid malignancy. Sci Rep. 2015;5:14869.

16. Chen M, Shen M, Li Y, Liu C, Zhou K, Hu W, et al. GC-MS-based metabolomic analysis of human papillary thyroid carcinoma tissue. Int J Mol Med. 2015;36(6):1607-14.

17. Yao Z, Yin P, Su D, Peng Z, Zhou L, Ma L, et al. Serum metabolic profiling and features of papillary thyroid carcinoma and nodular goiter. Mol Biosyst. 2011;7(9):2608-14.

18. Lim YJ, Xiu SG, Kuruvilla MS, Winquist E, Welch S, Black M, et al. Metabolomic identification of predictive and early biomarkers of cisplatin-induced acute kidney injury in adult head and neck cancer patients. Br J Clin Pharmacol. 2023.

19. Gao H, Song Y, Ma J, Zhai J, Zhang Y, Qu X. Untargeted metabolomics analysis of omeprazole-enhanced chemosensitivity to cisplatin in mice with non-small cell lung cancer. Chem Biol Interact. 2022;360:109933.

20. Huang H, van Dullemen LFA, Akhtar MZ, Faro ML, Yu Z, Valli A, et al. Proteo-metabolomics reveals compensation between ischemic and non-injured contralateral kidneys after reperfusion. Sci Rep. 2018;8(1):8539.

21. Cheng Y, Chen Y, Zhao M, Wang M, Liu M, Zhao L. Metabolomic profiling reveals the mechanisms underlying the nephrotoxicity of methotrexate in children with acute lymphoblastic leukemia. Pediatr Blood Cancer. 2023;14:e30578.

22. Verstraeten L, Den Abt R, Ghesquière B, Jochmans I. Current Insights into the Metabolome during Hypothermic Kidney Perfusion-A Scoping Review. J Clin Med. 2023;12(11):3613.
